# The origin of the lower fourth molar in canids, inferred by individual variation

**DOI:** 10.7717/peerj.2689

**Published:** 2016-11-08

**Authors:** Masakazu Asahara

**Affiliations:** College of Liberal Arts and Sciences, Mie University, Tsu, Mie, Japan

**Keywords:** Supernumerary molar, Dental anomaly, Dental formula, Inhibitory cascade

## Abstract

**Background:**

An increase in tooth number is an exception during mammalian evolution. The acquisition of the lower fourth molar in the bat-eared fox (*Otocyon megalotis*, Canidae, Carnivora, Mammalia) is one example; however, its developmental origin is not clear. In some canids (Canidae), individual variation exist as supernumerary molar M_4_. This study focuses on the acquisition of the lower fourth molar in canids and proposes that the inhibitory cascade model can explain its origin.

**Methods:**

Occlusal view projected area of lower molars was determined from 740 mandibles obtained from *Canis latrans*, *Nyctereutes procyonoides*, and *Urocyon cinereoargenteus* museum specimens. For each molar, relative sizes of molars (M_2_/M_1_ and M_3_/M_1_ scores) affected by inhibition/activation dynamics during development, were compared between individuals with and without supernumerary molar (M_4_).

**Results:**

Possession of a supernumerary molar was associated with significantly larger M_2_/M_1_ score in *Canis latrans*, M_3_/M_1_ score in *Nyctereutes procyonoides*, and M_2_/M_1_ and M_3_/M_1_ scores in *Urocyon cinereoargenteus* compared to individuals of these species that lacked supernumerary molars.

**Discussion:**

We propose that, in canids, the supernumerary fourth molar is attributable to reduced inhibition and greater activation during molar development. In the bat-eared fox, altered inhibition and activation dynamics of dental development during omnivorous-insectivorous adaptation may be a contributing factor in the origin of the lower fourth molar.

## Introduction

During the evolution of mammalian dentition, the number of teeth usually declines (Davit-Béal, Tucker & Sire, 2009; Ungar, 2010; Jernvall & Thesleff, 2012). While there are possible examples of secondary acquisition of recently lost teeth (M_3_ in callitrichine monkey: Scott, 2015), increased tooth number is a rare evolutionary event. Only whales (Cetacea), armadillos (Cingulata) and the bat-eared fox (*Otocyon megalotis*, Canidae, Carnivora) have evolved to increase the number of permanent teeth beyond the ancestral eutherian basic dental formula (I 3/3, C1/1, P 4/4 M 3/3) (Ungar, 2010). Some species, such as the manatee (*Trichechus*, Trichechidae, Sirenia), pigmy rock wallaby (*Petrogale concinna*, Macropodidae, Marsupialia), and silvery mole-rat (*Heliophobius argenteocinereus*, Bathyergidae, Rodentia), exhibit continuous horizontal replacement of teeth (Ungar, 2010; Gomes Rodrigues et al., 2011). However, only the bat-eared fox (*O. megalotis*) has shown an increase in tooth number, accompanied by neither morphological simplification nor continuous horizontal replacement. The dental formula in the bat-eared fox is I 3/3, C1/1, P 4/4 M 3–4/4–5, with these animal usually possessing an upper M^3^ and lower M_4_ that ancestral Canidae lacked (Sillero-Zubiri, 2009; Ungar, 2010), and this is considered a rare case of increased functional teeth number beyond that of the extant eutherian basic dental formula (Sillero-Zubiri, 2009; Ungar, 2010).

Bat-eared foxes are primarily insectivorous (Sillero-Zubiri, 2009), with a molar morphology comprising an undeveloped carnassial blade, equally sized molars (in relation to the other canids) and increased number of molars, which are attributable to an adaptation to an insectivorous diet (Wang & Tedford, 2008; Asahara, 2013; Asahara et al., 2016). It has been proposed that this dentition is suitable to a diet of insects that are small relative to the body size of the bat-eared fox, with a larger molar row grinding surface that enables greater chewing efficiency (Asahara, 2013; Asahara et al., 2016). However, this does not explain the presence of the fourth molar, the developmental origin of which remains unclear.

Individual variations in tooth number (supernumerary and missing teeth) have been reported in many mammals (e.g. Miles & Grigson, 1990). In some cases, as discussed by Asahara, Kryukov & Motokawa (2012), individual variations may underpin evolution, that is, fixation of the variation could initiate a new dental formula. In the present study, the source of the M_4_ in the bat-eared fox was investigated based on examination of supernumerary teeth in related species.

There are several reports of supernumerary molars in Canidae, including M^3^ and M_4_ along the normally aligned tooth row in coyote (*Canis latrans*) (Hall, 1940; Paradiso, 1966) and gray fox (*Urocyon cinereoargenteus*) (Hall, 1940). Wolsan (1984) categorized two types of supernumerary tooth generation: (1) creation of additional tooth germ and (2) splitting of a tooth germ. Type 1 can explain most of the supernumerary teeth that appear in positions where the ancestor possessed teeth, whereas type 2 can explain the eruption of supernumerary teeth that possess abnormal morphology relative to adjacent teeth. While the M^3^ in the coyote can be regarded as type 1 and “atavistic,” M_4_ is not readily explained in this manner. Although ancestral caniforms possessed M^3^ (Wang, 1994; Tomiya, 2011), the last ancestor possessing a functional M_4_ may be as early as the Mesozoic period, since the common ancestor of Placentalia possessed only three lower molars (O’Leary et al., 2013).

A previous experimental study established a developmental model termed the inhibitory cascade model (IC model) to explain relative molar size among three lower molars in mammals as resulting from the balance of inhibition and activation molecules during dental development (Kavanagh, Evans & Jernvall, 2007). In this model, activation molecules from the mesenchyme stimulate the formation of distal molars (M_2_ and M_3_), which makes them larger in relation to M_1_, whereas inhibition molecules secreted from M_1_ suppress distal molars growth (M_2_ and M_3_) (Kavanagh, Evans & Jernvall, 2007). According to the balance of these two factors, the relative size of M_1_, M_2_, and M_3_ (typically shown numerically as M_2_/M_1_ and M_3_/M_1_ size ratios; the size is defined as projected tooth area from occlusal view) result in a pattern of M_1_ > M_2_ > M_3_, M_1_ = M_2_ = M_3_, or M_1_ < M_2_ < M_3_ (Kavanagh, Evans & Jernvall, 2007). This model has previously been applied to explain dental variation in Carnivora (Polly, 2007; Halliday & Goswami, 2013; Asahara, 2013; Asahara et al., 2016).

The IC model can also explain the loss of M_3_ during murine evolution, such that greater inhibition and lower activation in experimental mice organs resulted in the disappearance of M_3_, coinciding with changes in the M_2_/M_1_ and M_3_/M_1_ ratios. This corresponds to the dentition of the murine species *Hydromys chrysogaster*, which lacks the M_3_ (Kavanagh, Evans & Jernvall, 2007). Asahara (2013) reported the relationship between dental anomalies (M_3_ loss) and the IC model in canids, in which M_2_/M_1_ scores of individuals that lost M_3_ were lower (indicating higher inhibition and lower activation) than the scores in normal individuals in local populations of raccoon dog (*Nyctereutes procyonoides*) and arctic fox (*Vulpes lagopus*) (Asahara, 2013). Evolutionary loss of M_3_ in murines and canids is thus considered attributable to inhibition/activation dynamics of dental development. Conversely, Kavanagh, Evans & Jernvall (2007) reported one case in which a supernumerary molar (M_4_) appeared in mice following suppression of inhibition molecules, indicative of an increase in molar number and altered inhibition/activation dynamics.

The working hypothesis for this study is that the fourth lower molars in the bat-eared fox and the supernumerary molar in some other canid species are generated by reduced inhibition and greater activation during dental development. This was tested by comparing the relative size of molars in locations of three canid species with some individuals possessing a supernumerary molar (M_4_).

## Materials and Methods

Mandible specimens (dentary bones and molars) of 451 *Canis latrans* (from the United States National Museum of Natural History), 153 *Nyctereutes procyonoides* (from the Primate Research Institute, Kyoto University, Japan), and 136 *Urocyon cinereoargenteus* (from the United States National Museum of Natural History) were examined. The collection of *C. latrans* was chosen based on an earlier study using the U.S. collection, which reported the presence of a supernumerary molar M_4_ in some specimens (Paradiso, 1966). Presence of the supernumerary molar was determined by macroscopic observation. Photographs were taken of the occlusal view of the molar row with scales. Projected areas of M_1_, M_2_, and M_3_ were manually measured using Image J software (NIH, Bethesda, MD, USA). The projected area is defined as molar size, as described by (Kavanagh, Evans & Jernvall, 2007; Asahara, 2013; Asahara, 2014). For specimens of *N. procyonoides*, most data were derived from Asahara (2014). The ratio of M_2_ to M_1_ size (M_2_/M_1_) and that of M_3_ to M_1_ size (M_3_/M_1_) were calculated to compare relative sizes among molars. According to the IC model (Kavanagh, Evans & Jernvall, 2007), lower inhibition and higher activation results in higher M_2_/M_1_ and M_3_/M_1_ scores, whereas increased inhibition and reduced activation lowers the M_2_/M_1_ and M_3_/M_1_ scores. M_1_ size and M_2_/M_1_ and M_3_/M_1_ scores of individuals with normal dentition, without M_3_, and with M_4_ were compared by U-test. Comparisons were performed separately for each species and sample location (as defined by collection source), with analyses performed using Minitab 14 statistical software (Minitab Inc., PA, USA). For data of the bat-eared fox *Otocyon megalotis* in Fig. 2 is cited from Asahara (2013).

## Results

**Figure 1:**
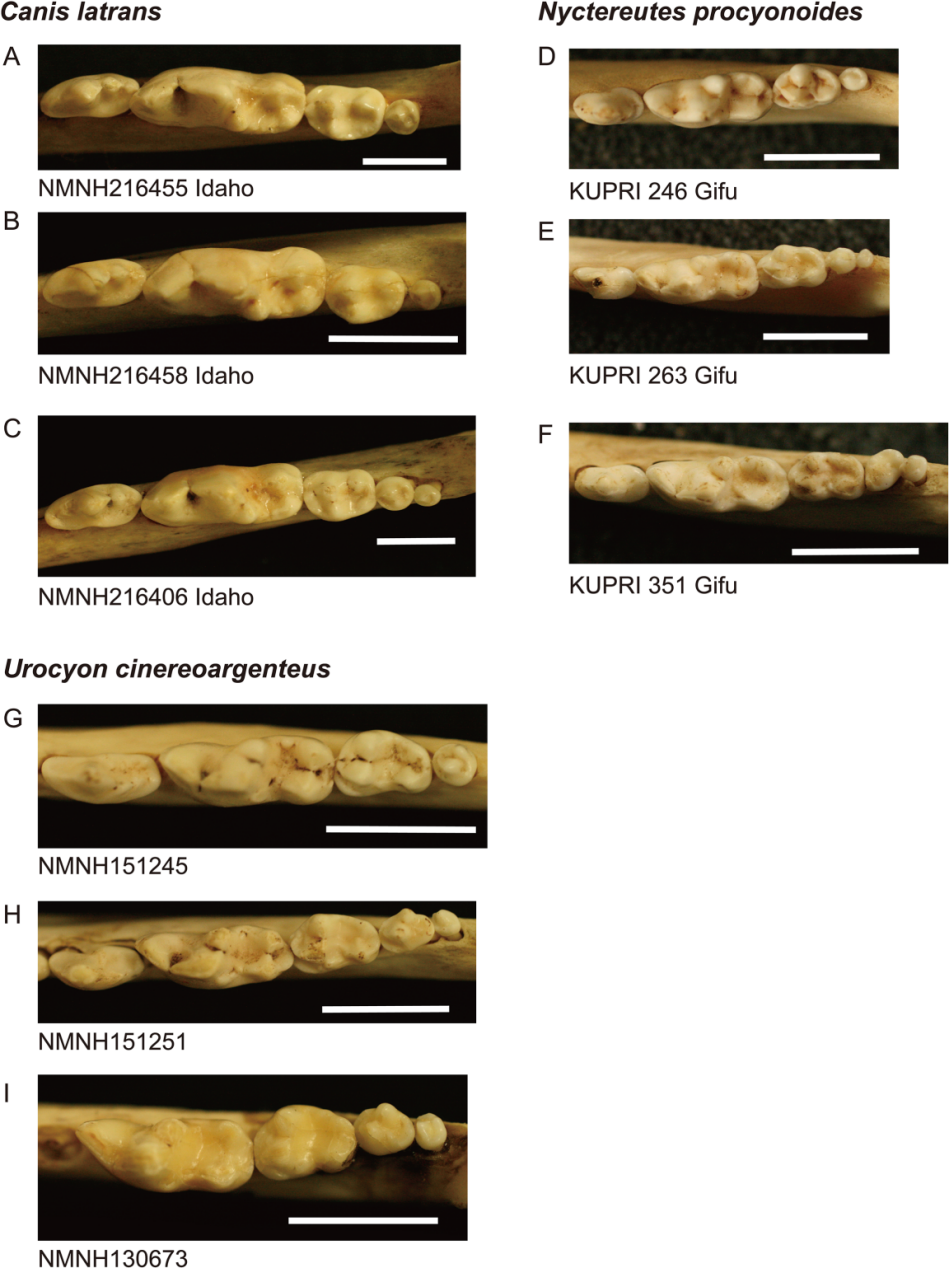
Images of lower molar rows (P_4_ to M_3_ or M_4_) from the occlusal view in the three species examined. (A–C): coyote *Canis latrans*, (D–F): raccoon dog *Nyctereutes procyonoides*, (G–I): gray fox *Urocyon cinereoargenteus*. Specimens A, B, D, and G possess normal dentition and specimens C, E, F, H, and I possess a supernumerary molar M_4_. Specimen numbers are depicted below the images. NMNH, the United States National Museum of Natural History, KUPRI, Primate Research Institute, Kyoto University. Scale: 10 mm.

**Figure 2:**
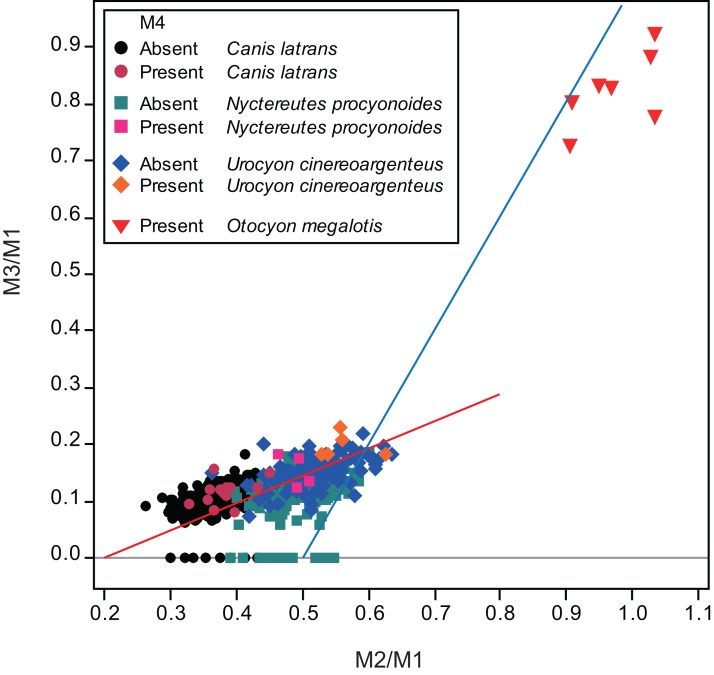
Bivariate plots of M_2_/M_1_ and M_3_/M_1_ scores of all specimens examined in this study and of the bat-eared fox *Otocyon megalotis* (Asahara, 2013). Specimens that do and do not possess M_4_ are depicted separately. The blue line indicates the molar ratio predicted by the inhibitory cascade model, as proposed by Kavanagh, Evans & Jernvall (2007), indicating variability found in molars of different murine species and experimentally generated mice. The red line indicates the trend of the molar ratio among canid species (Asahara, 2013).

**Figure 3:**
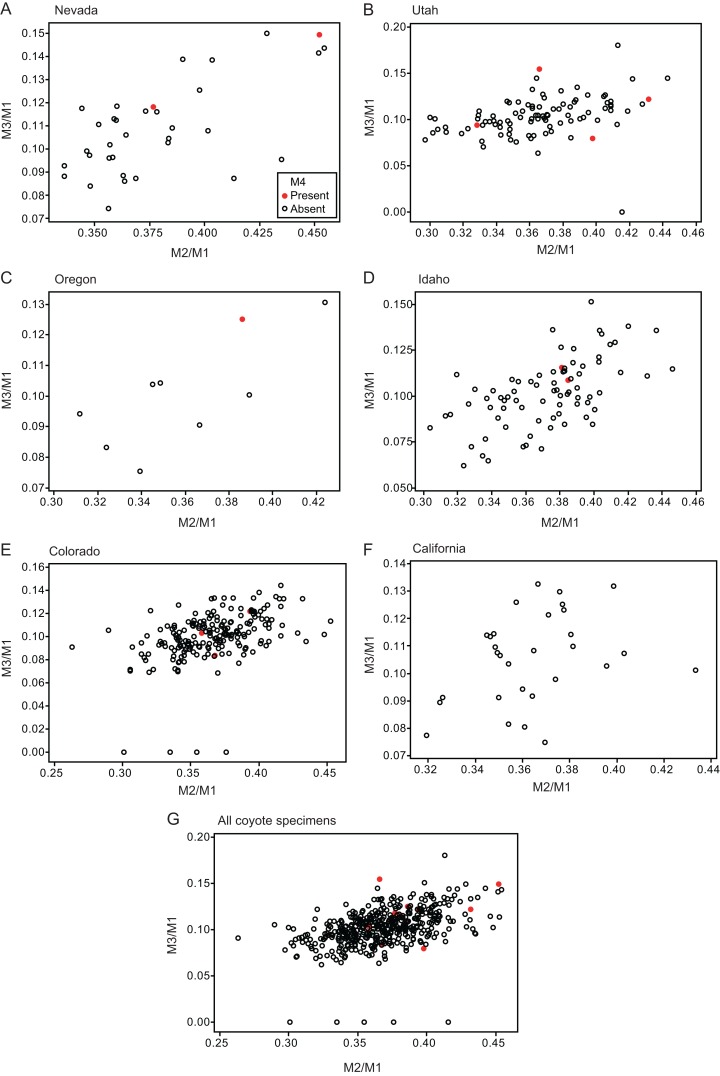
Bivariate plots of M_2_/M_1_ and M_3_/M_1_ scores among specimens of coyote *Canis latrans*. Specimens possessing M_4_ (red circle) and those lacking M_4_ (black circle) are shown separately.

**Figure 4:**
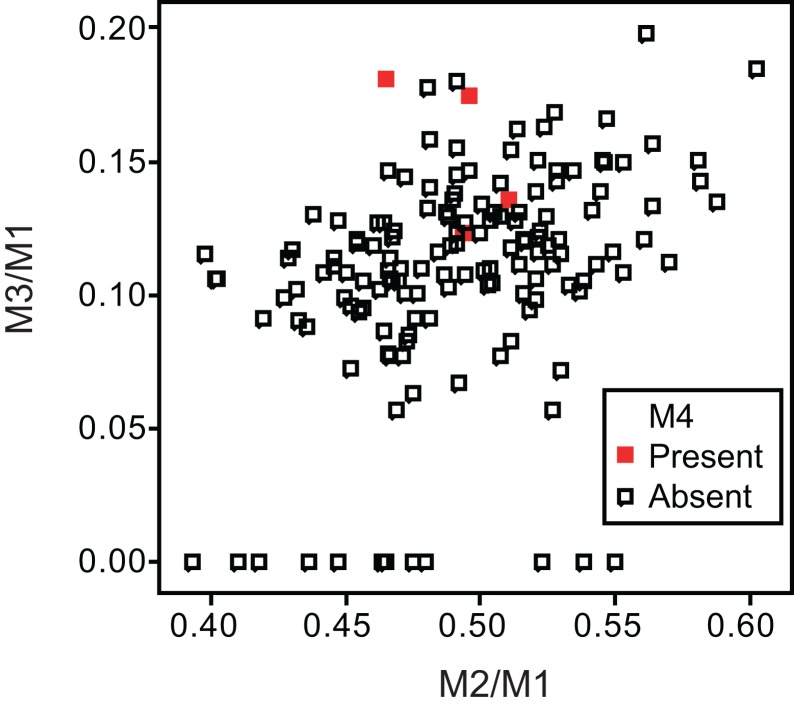
Bivariate plots of M_2_/M_1_ and M_3_/M_1_ scores among specimens of raccoon dog *Nyctereutes procyonoides*. Specimens possessing M_4_ (red square) and those lacking M_4_ (black square) are shown separately.

**Figure 5:**
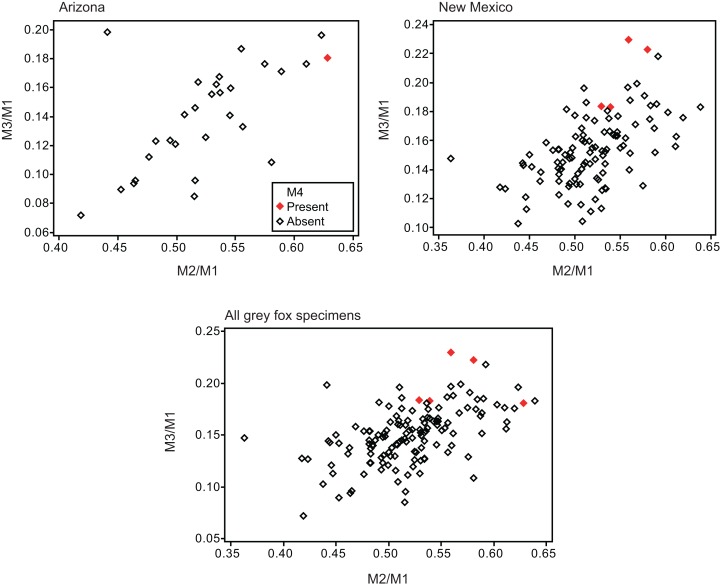
Bivariate plots of M_2_/M_1_ and M_3_/M_1_ scores among specimens of gray fox *Urocyon cinereoargenteus*. Specimens possessing M_4_ (red diamond) and those lacking M_4_ (black diamond) were shown separately.

Twelve individuals of *C. latrans* (2.66% of total 451 individuals), four individuals of *N. procyonoides* (2.61% of total 153 individuals), and five individuals of *U. cinereoargenteus* (3.68% of total 136 individuals) possessed an M_4_ in the normally aligned molar row (Fig. 1), with occurrences differing according to species and specimen location (Table 1). Data relating to M_1_ size and M_2_/M_1_ and M_3_/M_1_ scores are presented in Table 1 and Fig. 2. The distribution of specimens with normal dentition represented a typical pattern of interspecific variation among canids (Asahara, 2013) (Fig. 2). M_2_/M_1_ and M_3_/M_1_ scores per location and per species are also presented (Figs. 3–5).

**Table 1:** M_1_ size, M_2_/M_1_ and M_3_/M_1_ scores of locations examined. Normal and abnormal individuals presented separately. P-value of U-test between normal and abnormal individuals of each location are shown.

Species	Location	Dental anomaly	M_1_ size ± SD	U-test	M_2_/M_1_ ± SD	U-test	M_3_/M_1_ ± SD	U-test	N	%
*Canis latrans*	Nevada	Normal	141.00 ± 12.76		0.377 ± 0.032		0.108 ± 0.019		32	
		M_4_ present	138.17 ± 6.13	0.742	0.414 ± 0.053	0.200	0.134 ± 0.022	0.085	2	5.88
	Utah	M_3_ missing	136.03 ±		0.416 ±		±		1	1.04
		Normal	148.34 ± 14.56		0.364 ± 0.031		0.113 ± 0.033		91	
		M_4_ present	135.16 ± 15.08	0.117	0.381 ± 0.044	0.442	0.113 ± 0.033	0.746	4	4.17
	Oregon	Normal	133.94 ± 8.33		0.356 ± 0.036		0.098 ± 0.017		8	
		M_4_ present	126.23 ±		0.386 ±		0.125 ±		1	11.11
	Idaho	Normal	146.55 ± 12.50		0.372 ± 0.030		0.102 ± 0.018		77	
		M_4_ present	149.54 ± 15.61	0.791	0.383 ± 0.003	0.585	0.112 ± 0.005	0.255	2	2.53
	Colorado	M_3_ missing	167.94 ± 5.76	**0.008**	0.342 ± 0.032	0.149	±		4	1.97
		Normal	149.02 ± 13.51		0.366 ± 0.029		0.102 ± 0.015		196	
		M_4_ present	156.08 ± 22.90	0.617	0.376 ± 0.015	0.483	0.097 ± 0.021	0.452	3	1.48
	California	Normal	135.38 ± 12.51		0.364 ± 0.024		0.106 ± 0.016		30	
		M_4_ present	±		±		±		0	0.00
	Total	M_3_ missing	161.56 ± 15.12	**0.030**	0.357 ± 0.043	0.497	±		5	1.10
		Normal	146.63 ± 13.98		0.367 ± 0.030		0.103 ± 0.017		434	
		M_4_ present	142.54 ± 17.01	0.233	0.386 ± 0.032	**0.045**	0.113 ± 0.024	0.139	12	2.66
*Nyctereutes procyonoides*	Gifu	M_3_ missing	44.32 ± 3.12	0.495	0.466 ± 0.049	**0.022**	±		13	8.50
		Normal	43.79 ± 3.36		0.495 ± 0.040		0.118 ± 0.040		136	
		M_4_ present	44.20 ± 1.52	0.648	0.492 ± 0.019	0.840	0.153 ± 0.029	**0.022**	4	2.61
*Urocyon cinereoargenteus*	Arizona	Normal	46.54 ± 4.31		0.522 ± 0.050		0.138 ± 0.036		28	
		M_4_ present	39.88 ±		0.628 ±		0.181 ±		1	3.45
	New Mexico	Normal	47.05 ± 3.53		0.518 ± 0.046		0.152 ± 0.022		103	
		M_4_ present	47.48 ± 1.52	0.634	0.552 ± 0.023	0.075	0.205 ± 0.025	**0.002**	4	3.74
	Total	Normal	46.94 ± 3.70		0.519 ± 0.047		0.149 ± 0.026		131	
		M_4_ present	45.96 ± 3.64	0.777	0.567 ± 0.039	**0.022**	0.200 ± 0.024	**0.001**	5	3.68
*Otocyon megalotis*		M_4_ present (normal)	18.81 ± 1.44		0.979 ± 0.057		0.828 ± 0.065		7	

**Note:**

Boldface types indicate significance.

No relationship was found between mean M_1_ size and presence or absence of M_4_ (Table 1). When specimens of all locations were combined, mean M_2_/M_1_ and M_3_/M_1_ scores were typically larger in individuals with M_4_ than those with normal dentition (without M_4_), with the exception of M_2_/M_1_ scores in the Gifu samples of *N. procyonoides* (Table 1). When each location was analyzed separately, no significant difference in M_2_/M_1_ scores existed between individuals with M_4_ and with normal dentition; however, when all locations were pooled, individuals with M_4_ showed significantly higher M_2_/M_1_ scores than individuals with normal dentition in *C. latrans* and *U. cinereoargenteus* (Table 1). M_3_/M_1_ scores of individuals with M_4_ were significantly higher than those of individuals with normal dentition in the Gifu samples of *N. procyonoides* and the New Mexico samples of *U. cinereoargenteus* (Table 1). For the pooled samples of *U. cinereoargenteus*, M_3_/M_1_ scores of individuals with M_4_ were significantly higher than those of individuals with normal dentition (Table 1).

Five individuals of *C. latrans* and thirteen individuals of *N. procyonoides* lacked M_3_ (Table 1). M_1_ size of individuals without M_3_ is significantly larger than that of individuals with normal dentition in *C. latrans*. M_2_/M_1_ scores of individuals without M_3_ were significantly smaller than those of individuals with normal dentition in *N. procyonoides*.

## Discussions

The lack of a relationship between M_1_ size and individuals with M_4_ versus those with normal dentition indicates that absolute M_1_ size does not affect the generation of the M_4_. However, individuals with high M_2_/M_1_ and M_3_/M_1_ scores (and thus with relatively large M_2_s and M_3_s) did tend to possess a supernumerary molar M_4_ (Table 1; Figs. 2–5). While these scores did not differ significantly in most within-location analysis except for M_3_/M_1_ scores in the Gifu samples of *N. procyonoides* and the New Mexico samples of *U. cinereoargenteus* (Table 1), these results could be affected by the low number of samples that possessed M_4_. To overcome this limitation, analysis of combined sample-locations did establish that M_2_/M_1_ scores were larger in individuals with M_4_ than those with normal dentition in *C. latrans* and *U. cinereoargenteus* (Table 1). These results support the hypothesis that altered inhibition/activation dynamics (i.e. lower inhibition and higher activation) is likely to direct the formation of the M_4_ in these species.

Geographic variation can affect M_2_/M_1_ scores of pooled locations, such that locations with higher M_4_ incidence simply have a larger M_2_/M_1_ score. This appears not to be an issue in this study, for two reasons. Firstly, a larger M_2_/M_1_ score in a location is indicative of a genetic background supporting lower inhibition and higher activation during molar development. Secondly, the ranges of mean M_2_/M_1_ and M_3_/M_1_ scores among locations were not larger than the difference between mean scores of individuals possessing or lacking the M_4_ (Table 1). The data considered herein supports a causal relationship between inhibition and activation dynamics and the occurrence of M_4_.

While the existence of M_4_ correlates with high M_2_/M_1_ and M_3_/M_1_ scores, there were many individuals that exhibited high M_2_/M_1_ or M_3_/M_1_ scores but whose mandible did not possess M_4_ (Figs. 1–5), indicating that inhibition/activation dynamics are likely to be only one of the causes of generating an extra molar. This interpretation is supported by the experimental suppression of inhibition in a mouse model resulting in only one case of the appearance of an extra molar (Kavanagh, Evans & Jernvall, 2007). Acquisition of M_4_ is rare during evolution of placental mammals (Sillero-Zubiri, 2009; Ungar, 2010), and it is logical that several barriers to M_4_ generation exist. Furthermore, occasional occurrence of M_4_, albeit at a low rate, suggests that canids may possibly possess a genetic background that favors the generation of the M_4_, and this may explain the evolution of an additional molar in the bat-eared fox *O. megalotis*.

M_2_/M_1_ scores were larger in individuals without M_3_ than those with normal dentition in *N. procyonoides* (Table 1). The result accords with the proposed relationship between dental anomalies (M_3_ loss) and the IC model reported by Asahara (2013). Significant differences in M_1_ size found in *C. latrans* (Table 1) could be affected by the low number of samples including a large individual that lacked M_3_.

Consistent with Paradiso (1966), this study found that the presence or absence of M_4_ differed among locations of *C. latrans*. Furthermore, Gisburne & Feldhamer (2005) reported that M_4_ was not observed among 510 specimens of Illinois *U. cinereoargenteus*, contrasting with the finding from this study of the presence of M_4_ in a low proportion of Arizona and New Mexico gray foxes. Most studies of raccoon dog (*N. procyonoides*) have reported no M_4_ molar in Japanese samples (Hata, 1972; Harada et al., 1989; Asahi & Mori, 1980; Nozaki, 1984; collectively 664 individuals), with a notable exception published by Machida & Saito (1986), who reported an M_4_ in just one of 137 raccoon dogs from Saitama prefecture in Japan. Therefore, the presence of M_4_ in the three canid species examined in this study is considered a rare anomaly.

Asahara (2013) and Asahara et al. (2016) reported that the relative molar size was reflective of the diet among species of canids. The more carnivorous canid species (such as *Canis lupus*) possess low M_2_/M_1_ scores and the more omnivorous species (such as *N. procyonoides* or *U. cinereoargenteus*) have higher scores (Asahara, 2013; Asahara et al., 2016). In addition, the insectivorous hoary fox *Lycalopex vetulus* has higher M_2_/M_1_ and M_3_/M_1_ scores than almost all other omnivorous species, and the insectivorous bat-eared fox *O. megalotis* has higher M_2_/M_1_ and M_3_/M_1_ scores than all other canids, indicating very low inhibition and high activation during molar development (Asahara, 2013). According to this pattern, the evolution of the insectivorous diet is associated with molars that are of more equal size (between M_1_, M_2_, and M_3_) than the carnivorous or omnivorous species, with higher M_2_/M_1_ and M_3_/M_1_ scores reflecting low inhibition and high activation during molar development. The present study is consistent with the hypothesis that low inhibition and high activation is a cause of M_4_ generation. Therefore, M_4_ is considered to be an adaptive trait providing a larger total occlusal surface area to enable the canid to digest a large amount of insects, as discussed by Asahara (2013) and Asahara et al. (2016). It is hypothesized that the ancestral species began to consume an insectivorous diet, and it followed that a pattern of low inhibition and high activation during molar development evolved to generate more equally sized molars (between M_1_, M_2_, and M_3_) than its ancestor, providing an evolutionary force supporting this adaptation to the insectivorous diet. Then, the inhibition/activation pattern generates M_4_ by chance. M_4_ is also an adaptive trait for insectivorous diet providing larger occlusal surface area and greater grinding function, therefore, presence of M_4_ is naturally selected and fixed in the ancestral lineage of the bat-eared fox. While no fossil canid species has been to found to possess an M_4_, several cases of a small supernumerary M^3^ has been reported in an individual of the *Prototocyon* genus (Petter, 1964; Petter, 1973), which may be closely related to (or belong to) *Otocyon* (Van Valen, 1964; Petter, 1964; Petter, 1973; Wang & Tedford, 2008; Tedford, Wang & Taylor, 2009; Werdelin & Peigne, 2010). This supports the argument that this genus was a transitional stage in which molar number increased. Further discoveries from the fossil record will be important in revealing the evolutionary history of the fourth molar.

## Conclusions

The presence of a supernumerary molar M_4_ observed in several canid species is influenced by low inhibition and high activation during molar development, with the presence of M_4_ in the bat-eared fox *O. megalotis* originating from low inhibition and high activation due to evolutionary pressure relating to an insectivorous diet.

## Supplemental Information

10.7717/peerj.2689/supp-1 Supplemental Information 1 Raw data of molar sizes and exstence of supernumerary molars for each specimen.

## Additional Information and Declarations

### Data Deposition

The following information was supplied regarding data availability: The raw data has been supplied as Supplemental Dataset Files.
